# Association between hyperkalemia, RAASi non-adherence and outcomes in chronic kidney disease

**DOI:** 10.1007/s40620-021-01070-6

**Published:** 2021-06-11

**Authors:** Antonio Santoro, Valentina Perrone, Elisa Giacomini, Diego Sangiorgi, Davide Alessandrini, Luca Degli Esposti

**Affiliations:** 1 UOC Nephrology, Dialysis and Hypertension, Scuola di Specializzazione in Nefrologia, Università degli studi di Bologna, Policlinico S.Orsola-Malpighi, Azienda Ospedaliero-Universitaria, Via P. Palagi, 9, Bologna, Italy; 2 CliCon S.r.l. Health, Economics and Outcomes Research, Bologna, Italy

**Keywords:** Chronic kidney disease, Potassium, Renin–angiotensin–aldosterone system, RAASi, Hyperkalemia, Cardiovascular events

## Abstract

**Background:**

Hyperkalemia is relatively frequent in CKD patients treated with renin-angiotensin-aldosterone-system inhibitors (RAASi).

**Aim:**

The aim of the present study was to estimate the increased risk of cardiovascular events and mortality due to sub-optimal adherence to RAASi in CKD patients with hyperkalemia.

**Methods:**

An observational retrospective cohort study was conducted, based on administrative and laboratory databases of five Local Health Units. Adult patients discharged from the hospital with a diagnosis of CKD, who were prescribed RAASi between January 2010 and December 2017, were included. We evaluated the appearance of documented episodes of hyperkalemia, RAASi therapy adherence and the effects of these two variables on cardiovascular events, death and dialysis inception for study patients.

**Results:**

Of the 9241 selected patients, 4451 met all the criteria for study inclusion. Among them, 1071 had at least one documented episode of hyperkalemia, while 3380 did not. After propensity score matching based on several variables we obtained 2 groups of patients. The appearance of hyperkalemia caused treatment discontinuation in 21.8% of patients previously on RAASi therapy, and sub-optimal adherence (proportion of days covered  < 80%) in 33.6% of them. Non-adherence to RAASi therapy among hyperkalemia patients was associated with a higher risk of cardiovascular events (hazard ratio [HR] 1.45, confidence interval [CI] 1.02–2.08; p < 0.05). Moreover, in non-adherent hyperkalemia patients, the risk of death increased by 126% (HR 2.26, CI 1.62–3.15; p < 0.001) compared with adherent patients.

**Conclusions:**

In a large cohort of CKD patients treated with RAASi, we observed that following hyperkalemia onset, non-adherence to RAASi medication can result in an increased risk of cardiovascular events and death.

**Graphical abstract:**

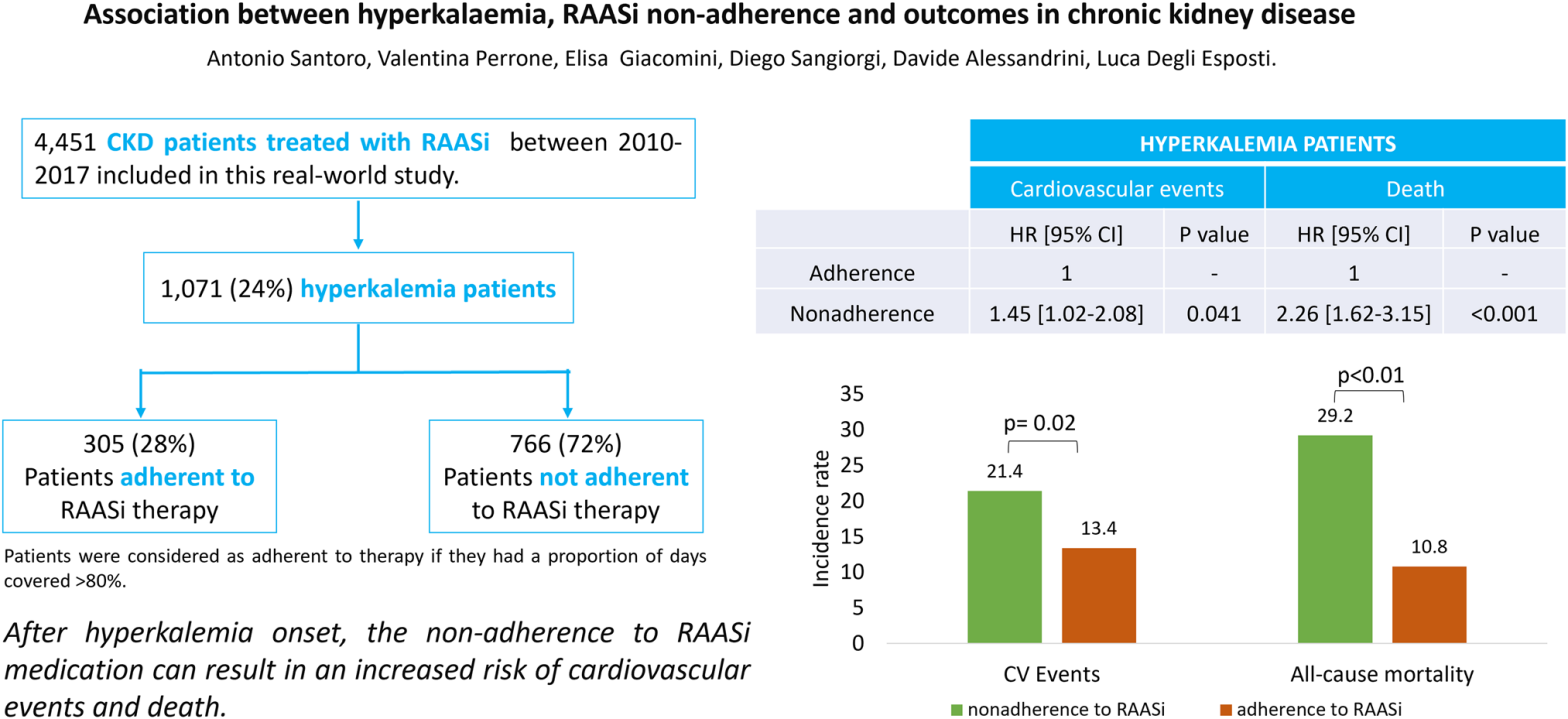

**Supplementary Information:**

The online version contains supplementary material available at 10.1007/s40620-021-01070-6.

## Introduction

Angiotensin converting enzyme inhibitors (ACEi) and angiotensin receptor blockers (ARBs) have demonstrated real efficacy in reducing blood pressure and proteinuria, and in slowing the progression of chronic kidney disease (CKD) [1–3]. Moreover, these drugs induce clinical improvement in patients with heart failure, diabetes mellitus and ischemic heart disease. However, this class of drugs has also been associated with adverse events, sometimes severe, such as the appearance of acute renal failure, severe hyperkalemia and significant reductions in blood pressure [4, 5]. Fear of adverse effects of renin–angiotensin–aldosterone-system inhibitors (RAASi), leads to either under-utilization or under-dosing, particularly in patients with multi-morbidity. A Turkish study evaluated the barriers limiting the use of ACEi and ARBs in patients with chronic renal failure and found that hyperkalemia is the main cause leading to the discontinuation of RAASi [6]. Shirazian et al. also showed that hyperkalemia is the main cause of under-utilization of ACEi and ARBs [7]. In order to avoid an unexpected increase in potassium, the Kidney Disease Improving Global Outcomes (KDIGO) guidelines [8] recommend evaluating the glomerular filtration rate (GFR) and serum potassium levels within a week of starting or increasing the dose of ACEi and ARBs, regardless of the basal potassium level. The American Heart Association made the same type of recommendation [9]. The Kidney Disease Outcomes Quality Initiative (KDOQI) suggests a more detailed algorithm, with monitoring of potassium levels 4 weeks after the beginning of RAASi therapy or after the dose increase, in patients at higher risk (defined as patients with systolic pressure below 120 mmHg, potassium > 4.5 mmol/l or a GFR < 60 ml/min) and, in any case, between 4 and 12 weeks in all other patients [1]. Hyperkalemia is common in CKD patients due to both the effects of kidney dysfunction on K^+^ homeostasis and to a cluster of co-morbidities [10]. RAASi may exacerbate hyperkalemia in CKD patients, with subsequent adverse outcomes including cardiovascular events [11]. However, studies assessing the incidence of hyperkalemia, specifically in patients with CKD receiving RAASi treatment, are limited. Furthermore, few data are available in the literature regarding the increased risk of negative outcomes related to hyperkalemia in patients on therapy with RAASi. In a prospective study, involving a cohort of non-dialysis CKD patients, Provenzano et al. [12], found that patients with hyperkalemia, who were not being treated or had discontinued previous RAASi therapy, had a 57% increased risk of reaching end stage renal disease (ESRD) compared to patients without hyperkalemia who had continued appropriate therapy with RAASi. However, to date, no study has evaluated the risk of cardiovascular events, mortality and kidney death from the under-use of RAASi in patients with hyperkalemia in the same real-world population and therefore outside of studies on selected populations.

The main aim of the present study was to estimate the increased risk of cardiovascular and mortality events due to sub-optimal adherence to RAASi in hyperkalemia-CKD patients. We also evaluated the increased risk of cardiovascular events, mortality and risk of dialysis inception in patients with CKD who developed hyperkalemia while on therapy with RAASi.

## Methods

### Data source

This observational retrospective cohort study was conducted by integrating the administrative and laboratory databases of five Local Health Units geographically distributed throughout Italy, which account for approximately 5% of the total Italian population. Data were collected from the following databases: beneficiaries’ database for demographic data; pharmaceuticals database to retrieve information related to drugs reimbursed by the Italian National Health Service (INHS), including anatomical-therapeutic chemical code (ATCC), number of packages, number of units per package, unit cost per package, and prescription date; hospitalization database providing all hospitalization data including admission and discharge dates, primary and secondary discharge diagnoses recorded according to the International Classification of Diseases, Ninth Revision, Clinical Modification (ICD-9-CM); outpatient specialist services database, which contains information on visits, services, laboratory exams and instrumental diagnostics exams provided in outpatient settings and reimbursed by the INHS; laboratory analysis registries that (allow to) collect data on potassium levels.

To guarantee the patients’ privacy, an anonymous univocal numeric code was assigned to each study subject, in full compliance with the European General Data Protection Regulation (GDPR) (2016/679). No identifiers related to patients were provided to the authors. The patient code in each database allowed electronic linkage among the databases. All the results of the analyses were produced as aggregated summaries, which cannot be assigned, either directly or indirectly, to individual patients. Informed consent was not required to use encrypted retrospective information for research purposes. On the basis of the Italian law regarding the conduction of observational analyses [13], the Local Ethics Committee of each participating Local Health Unit was notified of this study and approved it.

### Cohort definition

All patients above 18 years of age were screened for eligibility if they had been discharged from hospital with an ICD-9-CM diagnosis code for CKD (585) from January 1st, 2010 to December 31st, 2017 (enrollment period). The date corresponding to the first hospitalization discharge related to CKD was considered the index date, and all patients had been characterized in the year prior to the index date. Among them, only patients that were prescribed RAASi (ATCC: C09) within three months of the index date were included in the analysis. The RAASi that were taken into consideration included; (a) ACEi—plain (ATCC: C09A), (b) ACEi—combinations (ATCC: C09B) and more specifically, ACEi and diuretics (ATCC: C09BA), ACEi and calcium channel blockers (ATCC: C09BB), ACEi—other combinations (ATCC: C09BX), (c) angiotensin II antagonists—plain (ATCC: C09C), (d) angiotensin II antagonist combinations (ATCC: C09D), (e) other agents acting on the renin-angiotensin system (ATCC: C09X), and more specifically, renin-inhibitors such as remikiren (ATCC: C09XA01, aliskiren (ATCC: C09XA02), aliskiren and hydrochlorothiazide (ATCC:C09XA52), aliskiren and amlodipine(ATCC: C09XA53), aliskiren, amlodipine and hydrochlorothiazide(ATCC: C09XA54). Patients were followed up from index date to December 31st, 2017. Subjects were excluded if they had a concomitant diagnosis of heart failure (ICD-9-CM code: 428), so that RAASi therapy could be reliably related to CKD disease, or if they were on dialysis (ICD-9-CM code: V560), or if the results of serum potassium level tests were not available. Moreover, patients who transferred to a different Local Health Unit during the study period were also excluded from the analysis.

### Study variables

Serum potassium levels were tested during the three months before and after the index date; hyperkalemia was ascertained by a serum potassium level ≥ 5.5 mmol/l, and hyperkalemia onset was defined as the first serum potassium level ≥ 5.5 mmol/l observed.

Adherence to RAASi therapy was evaluated in the 12-months after the index date by calculating the proportion of days covered (PDC), i.e. the ratio between the number of days of medication and days of observation (365 days), multiplied by 100. Patients were considered as being adherent to therapy if they had a PDC > 80%. PDC is considered one of the most reliable methods for measuring adherence in chronic therapies, and a standard threshold of 80%, which represents the likelihood of achieving the most clinical benefit, is widely accepted [14, 15].

Information concerning demographic and clinical characteristics, as well as previous RAASi (ATCC: C09) prescription and hospitalization related to CKD during the characterization period were collected at baseline. Previous treatments that were analyzed included diuretic drugs (ATCC: C03 except for code C03DA), aldosterone antagonists (ATCC: C03DA), beta blocking agents (ATCC: C07), lipid modifying agents (ATCC: C10), and anti-diabetic agents (ATCC: A10). CKD stages were identified as follows: CKD stage ≤ 3 by ICD-9-CM code 585.1, 585.2, 585.3; CKD stage > 3 by ICD-9-CM code: 585.4, 585.5; patients with ICD-9-CM code 585.9 were classified as “not specified” and were not considered for multivariable analyses.

Comorbidities were measured using the charlson comorbidity index [16] that assigns a weighted score to each concomitant disease identified through the discharge diagnoses/pharmaceutical prescriptions recorded during the characterization period.

Events that were analyzed during follow-up included: cardiovascular events [acute myocardial infarction (ICD-9-CM code: 410), other acute and sub-acute forms of ischemic heart disease (ICD-9-CM code: 411), angina pectoris (ICD-9-CM code: 413), other forms of chronic ischemic heart disease (ICD-9-CM code: 414), cerebrovascular disease (ICD-9-CM code: 430–438), atherosclerosis (ICD-9-CM code: 440) and other peripheral vascular disease (ICD-9-CM code: 443)], dialysis (ICD-9-CM code: V560) and death. For the estimation of the hazard ratio (HR), patients that experienced such events in the three months after the index date were excluded.

### Statistical analysis

Continuous variables were reported as mean ± standard deviation (SD); categorical variables were expressed as numbers and percentages. In both cohorts, clinical and demographic characteristics were evaluated and compared among patients with and without hyperkalemia. Given the non randomized nature of the study, to minimize selection bias, a second multivariable analysis was performed using propensity score matching (PSM), that was determined using a logistic regression model from which the probability of being hyperkalemic or non-hyperkalemic was calculated for each patient. Variables included in the model were age, gender, Charlson index, CKD stage and presence of treatment (RAASi, diuretics, aldosterone antagonists, beta blocking agents, lipid modifying agents, anti-diabetics) or hospitalization prior to the index date. Model discrimination was assessed using the C statistic (0.68 for hyperkalemia vs non-hyperkalemia patients and 0.57 for adherence vs non-adherence), and model calibration using the Hosmer–Lemeshow test (0.43 for hyperkalemia vs non-hyperkalemia patients and 0.23 for adherence vs non-adherence).

Incidence rates were calculated as the total number of events per 100 person-years in each group. Cox proportional hazard model was used to estimate HR with relative 95% confidence interval (CI) before and after PSM; the proportional hazards assumption was tested using Schoenfeld residuals. The Cox model, considering the population pre-PSM is published elsewhere [17]. In the present study, we report the analysis performed after PSM considering the aforementioned potentially confounding variables. Moreover, regarding the pre-PSM cohort, we report the Cox proportional hazard model adjusted for the same variables. Schoenfeld scaled and unscaled residuals were analyzed in order to assess the proportional hazards assumption.

Student’s T test was used to compare continuous variables, and the chi square test was used for categorical ones. Statistical significance was accepted at p < 0.05. All analyses were performed using STATA SE version 12.0.

## Results

**Fig. 1:**
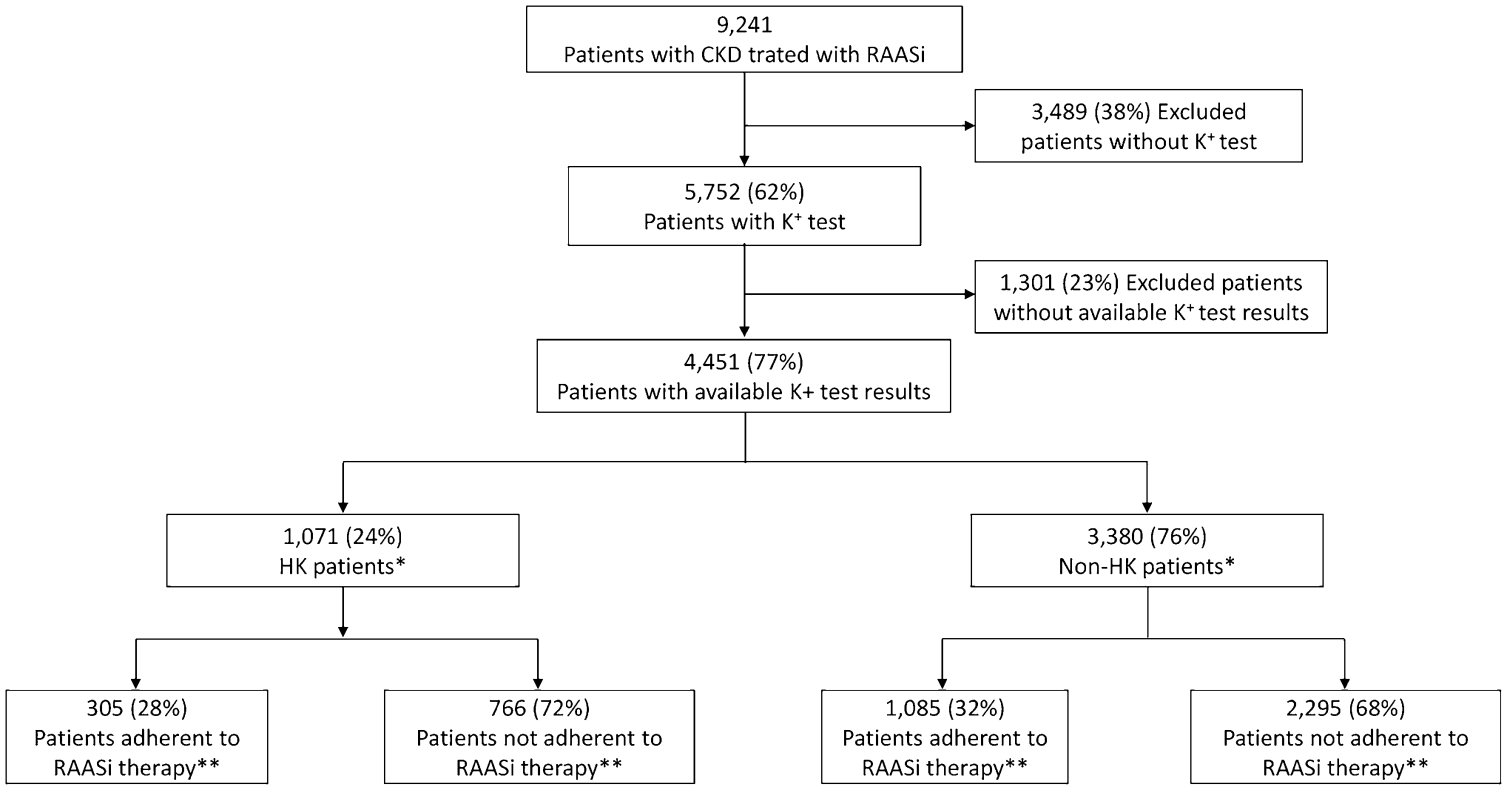
Flow-chart of included patients. *Patients were considered as having hyperkalemia if they presented a serum potassium level ≥ 5.5 mmol/l. **Patients were considered as adherent to therapy if they had a proportion of days covered (PDC) > 80%. *CKD* chronic kidney disease, *HK* hyperkalemia, *RAASi* renin–angiotensin–aldosterone-system inhibitor

In the present analysis, 9241 CKD patients treated with RAASi were screened for eligibility. After applying the exclusion criteria, as reported in Fig. 1, 4451 patients were included in the study: among them, 1071 (24%) had hyperkalemia (hyperkalemia patients), while 3380 (76%) did not (non-hyperkalemia patients).

Demographic and clinical characteristics of hyperkalemia and non-hyperkalemia patients, at baseline before and after PSM, are reported in Table 1. Hyperkalemia patients had a mean age of 72.8 ± 13.8 years, younger than non-hyperkalemia patients (mean age 75.0 ± 13.1 years) (p < 0.001). In both groups, the majority of patients were male (58.3% hyperkalemia, 60.4% non-hyperkalemia). Concerning the severity of CKD, early CKD stages (CKD1 to 3) were more frequent in non-hyperkalemia than in hyperkalemia patients (67.5% vs 51.4%; p < 0,001), while later stages (CKD4 to 5) were more frequent in hyperkalemia patients (37.8% vs 17.7%; p < 0,001). In both groups, 92% of patients had a previous medical prescription for RAASi. In hyperkalemia patients, non-adherence to RAASi therapy was more frequent (N = 766, 72%) than adherence (N = 305, 28%) (Fig. 1). Hospitalizations prior to the index date were observed in 4.6% of hyperkalemia patients and in 2.2% of non-hyperkalemia patients (p < 0.001). Other previous treatments that were identified during the characterization period are reported in Table 1. Furthermore, after PSM analysis, these differences in demographic and clinical characteristics between hyperkalemia and non-hyperkalemia patients were no longer statistically significant (Table 1).

**Table 1:** Demographic characteristics, previous treatments and comorbidities of included patients before and after PSM

	Before PSM			After PSM		
	HK pt	Non-HK pt	p	HK pt	Non-HK pt	p
N	1071	3380		881	881	
Age, mean ± SD	72.8 ± 13.8	75.0 ± 13.1	< 0.001	71.8 ± 14.3	72.9 ± 14.1	0.104
Male, n (%)	624 (58.3)	2,043 (60.4)	0.204	514 (58.3)	500 (56.8)	0.500
Charlson comorbidity index, mean ± SD [median, IQR range 1–3]	2.19 ± 1.77 [2, 1–3]	1.88 ± 1.63 [2, 1–3]	< 0.001	2.14 ± 1.76 [2, 1–3]	2.07 ± 1.81 [2, 1–3]	0.411
CKD stage, n (%)						
≤ 3	551 (51.5)	2280 (67.5)	< 0.001	501 (56.9)	511 (58.0)	0.665
> 3	405 (37.8)	599 (17.7)		380 (43.1)	370 (42.0)	
Not specified	115 (10.7)	501 (14.8)	–			
Previous RAASi use, n (%)	985 (92.0)	3112 (92.1)	0.915	813 (92.3)	818 (92.8)	0.650
Previous hospitalization related to CKD, n (%)	49 (4.6)	73 (2.2)	< 0.001	44 (5.0)	38 (4.3)	0.497
Previous drug treatments						
Diuretics, n (%)	684 (63.9)	1793 (53.0)	< 0.001	570 (64.7)	569 (64.6)	0.960
Aldosterone antagonists, n (%)	143 (13.4)	302 (8.9)	< 0.001	121 (13.7)	121 (13.7)	1.000
Beta blocking agents, n (%)	442 (41.3)	1436 (42.5)	0.483	364 (41.3)	378 (42.9)	0.499
Lipid modifying agents, n (%)	539 (50.3)	1722 (50.9)	0.724	449 (51.0)	453 (51.4)	0.849
Anti-diabetics, n (%)	473 (44.2)	1198 (35.4)	< 0.001	394 (44.7)	382 (43.4)	0.565

*PSM* propensity score matching, *HK* hyperkalemia, *pt* patients, *CKD* chronic kidney disease, *RAASi* renin–angiotensin–aldosterone-system inhibitor, *SD* standard deviation

Appearance of hyperkalemia led to treatment discontinuation in 21.8% of patients who previously adhered to RAASi therapy, and to sub-optimal adherence (PDC < 80%) in 33.6% of them, while only 44.6% remained adherent to their prescribed RAASi therapy.

**Fig. 2:**
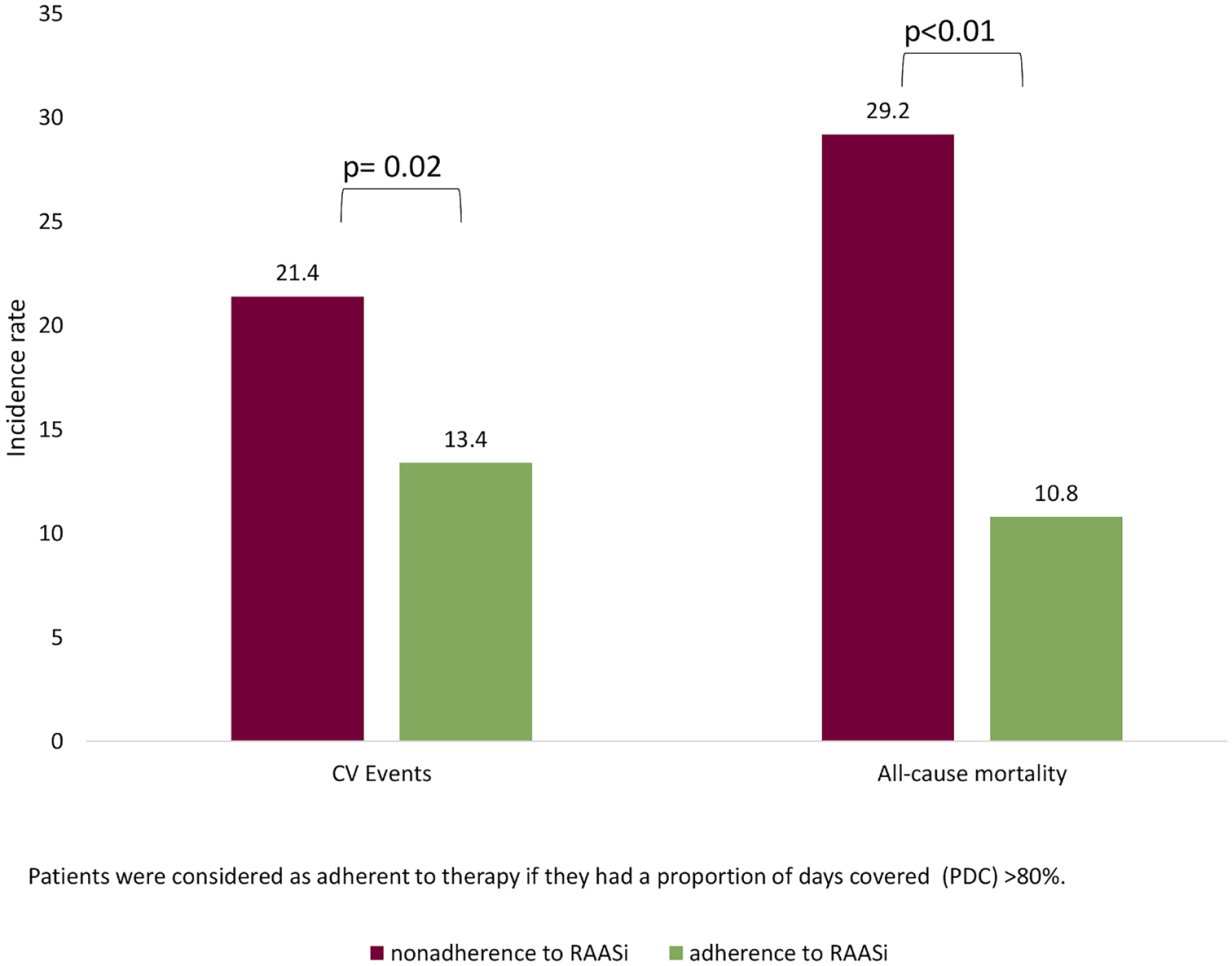
Incidence rate/100 person-years of cardiovascular events or all-cause mortality for non-adherent vs adherent to RAASi patients. Patients were considered adherent to therapy if they had a proportion of days covered (PDC) > 80%

As shown in Table 2, after PSM, non-adherence to RAASi therapy among hyperkalemia patients was associated with a higher risk of cardiovascular events (HR 1.45, CI 1.02–2.08; p < 0.05). Moreover, in non-adherent hyperkalemia patients, the risk of death increased by 126% (HR 2.26, CI 1.62–3.15; p < 0.001) compared to adherent patients. The incidence of cardiovascular events was higher among hyperkalemia non-adherent patients (21.4/100 person-years) than in adherent ones (13.4/100 person-years). Similarly, the all-cause mortality rate reached 29.2/100 person-years in non-adherent patients, compared to 10.8/100 person-years in adherent patients (Fig. 2). In the Cox adjusted model, comparable results were obtained (Supplementary Table 1). A further analysis examined the association of non-adherence with negative outcomes among non-hyperkalemia patients (Supplementary Table 3); in this cohort non-adherence was significantly associated with the risk of death (HR 1.29, CI 1.07–1.56; p 0.009), which was however lower compared to the hyperkalemia cohort.

**Table 2:** Risk of cardiovascular events or death for non-adherent vs adherent to RAASi hyperkalemia patients after PSM

	Cardiovascular events		Death	
	HR [95% CI]	p value	HR [95% CI]	p value
Adherence	1	–	1	–
Nonadherence	1.45 [1.02–2.08]	0.041	2.26 [1.62–3.15]	< 0.001
Age	1.01 [1.00–1.03]	0.032	1.05 [1.04–1.07]	< 0.001
Male gender	1.99 [1.34–2.96]	0.001	1.05 [0.75–1.47]	0.783
Charlson comorbidity index	0.98 [0.88–1.09]	0.687	1.21 [1.11–1.32]	< 0.001
CKD stage	0.80 [0.55–1.14]	0.218	1.10 [0.79–1.53]	0.576

Due to the low sample size, only age, male gender, CCI and CKD stage were considered

*CKD* chronic kidney disease, *PSM* propensity score matching, *HR* hazard ratio, *CI* confidence interval

**Fig. 3:**
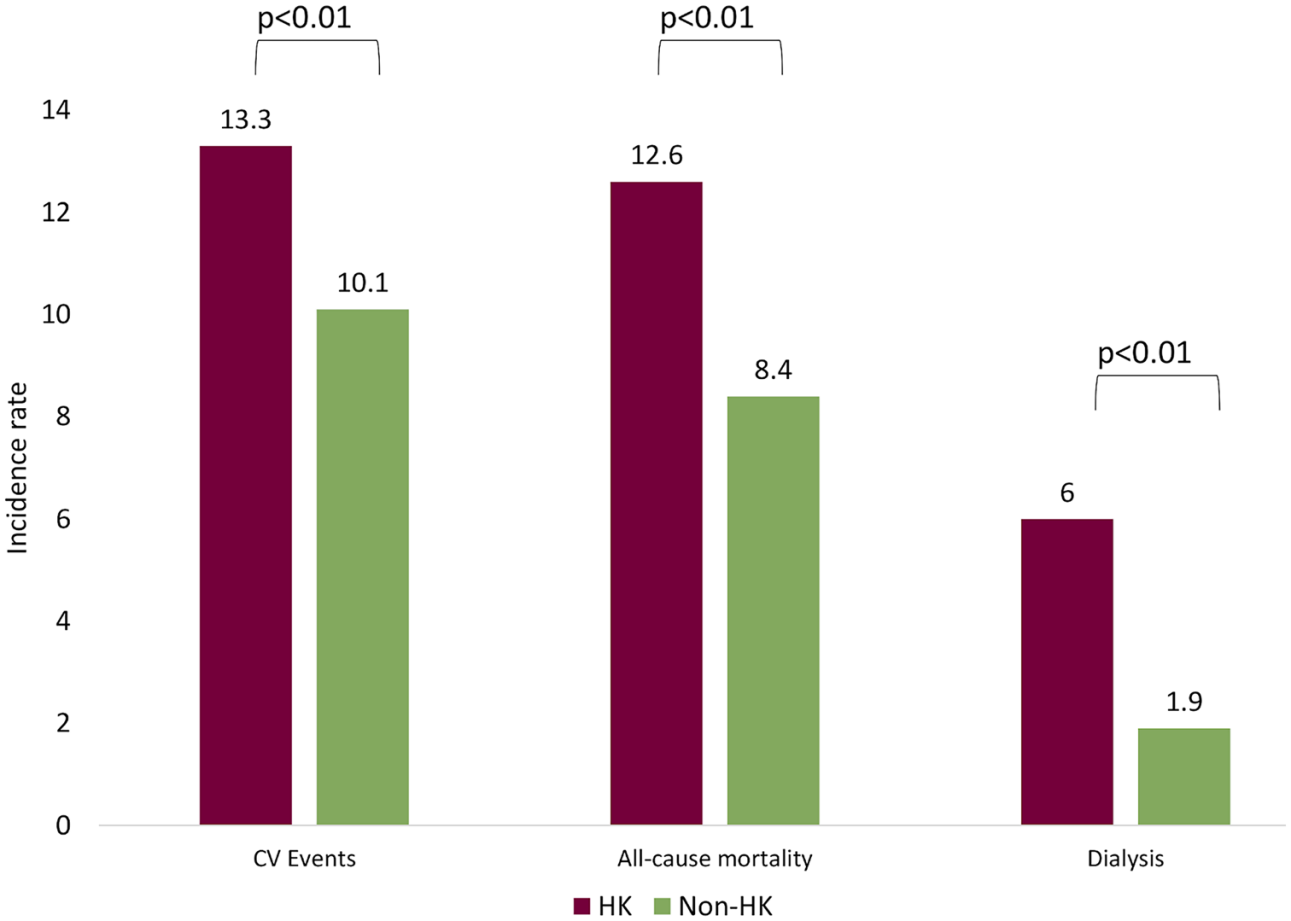
Incidence rate/100 person-years of CV events, all-cause mortality or dialysis for hyperkalemia vs non-hyperkalemia patients. *CV* cardiovascular, *HK* hyperkalemia

In further analyses, HR between hyperkalemia and non-hyperkalemia for cardiovascular events, death or dialysis was evaluated after PSM, as reported in Table 3. After PSM, hyperkalemia patients showed an increased risk of cardiovascualr events (HR 1.34, CI 1.13–1.59; p < 0.001), death (HR 1.53, CI 1.29–1.80; p < 0.001) and dialysis inception (HR 3.16, CI 2.29–4.37; p < 0.001). Cardiovascular events also seemed to be related, at least on the basis of statistical significance, to age, male gender, and the use of beta-blockers, lipid modifying agents and anti-diabetics. The risk of death would appear to increase with age, Charlson index, CKD stage, and the use of RAASi, diuretics, and lipid modifying agents. CKD stage (HR 4.81, CI 3.34–6.92; p < 0.001) and previous hospitalizations related to CKD, seem to influence the need for dialysis. On the contrary, older age may result as a protective factor vs. dialysis initiation. The Cox adjusted model found similar results 8 (Supplementary Table 2). As shown in Fig. 3, the incidence rate for cardiovascular events was 13.3/100 patient-years in hyperkalemia patients vs 10.1 in non-hyperkalemia patients (p < 0.01, median time to event 1.9 years), while for all-cause mortality it was 12.6 vs 8.4 (p < 0.01, median time to death 2.7 years), and for dialysis it was 6 vs 1.9 (p < 0.01, median time to dialysis 2.3 years).

**Table 3:** Risk of cardiovascular events, death or dialysis for hyperkalemia vs non-hyperkalemia patients after PSM

	Cardiovascular events		Death		Dialysis	
	HR [95% CI]	p value	HR [95% CI]	p value	HR [95% CI]	p value
Non-hyperkalemia	1	–	1	–	1	–
Hyperkalemia	1.34 [1.13–1.59]	< 0.001	1.53 [1.29–1.80]	< 0.001	3.16 [2.29–4.37]	< 0.001
Age	1.02 [1.01–1.03]	< 0.001	1.06 [1.05–1.07]	< 0.001	0.97 [0.96–0.98]	< 0.001
Male gender	1.43 [1.19–1.71]	< 0.001	1.04 [0.88–1.23]	0.629	0.94 [0.70–1.25]	0.658
Charlson comorbidity index	1.01 [0.96–1.07	0.664	1.19 [1.13–1.24]	< 0.001	0.91 [0.81–1.02]	0.103
CKD stage	1.13 [0.94–1.35]	0.183	1.38 [1.16–1.63]	< 0.001	4.81 [3.34–6.92]	< 0.001
Previous hospitalization related to CKD	0.90 [0.63–1.27]	0.534	0.88 [0.63–1.24]	0.467	2.26 [1.49–3.44]	< 0.001
Previous drug treatments						
RAASi use	0.86 [0.61–1.23]	0.413	0.72 [0.52–0.99]	0.042	0.73 [0.46–1.16]	0.184
Diuretics (%)	1.12 [0.92–1.37]	0.256	1.30 [1.07–1.59]	0.009	0.85 [0.62–1.18]	0.335
Aldosterone antagonists	0.99 [0.77–1.27]	0.916	1.10 [0.88–1.37]	0.416	0.53 [0.28–0.98]	0.043
Beta blocking agents	1.22 [1.02–1.46]	0.028	0.94 [0.79–1.12]	0.501	1.12 [0.82–1.52]	0.485
Lipid modifying agents	1.70 [1.41–2.05]	< 0.001	0.79 [0.66–0.94]	0.007	1.27 [0.94–1.73]	0.126
Anti-diabetics	1.26 [1.03–1.54]	0.025	1.00 [0.83–1.21]	0.977	1.34 [0.93–1.93]	0.122
RAASi dosage	1.00 [1.00]	0.152	1.00 [1.00]	0.194	1.00 [1.00]	0.867

*PSM* propensity score matching, *HR* hazard ratio, *CI* confidence interval

## Discussion

We evaluated the simultaneous risk of cardiovascular events and mortality from the under-use of RAASi in patients with hyperkalemia in a real-world CKD population. In this large population-based study, we have shown that non-adherence to RAASi therapy leads to a greater risk of cardiovascular events and death. Furthermore, patients with hyperkalemia present a significantly increased risk of cardiovascular events, all-cause mortality and dialysis onset. We have already reported these results [17] and they were confirmed in the pre-PSM cohort even when the model was adjusted for potentially confounding variables. Almost one-fourth of the patients (24%) in our study had a serum potassium level ≥ 5.5 mmol/l. Patients with CKD (especially advanced CKD) are at high risk for hyperkalemia, particularly when other factors hampering renal potassium excretion and comorbidities are present [11]. Hyperkalemia prevalence in CKD patients is considerably higher than in the general population. A recent review reports a hyperkalemia frequency as high as 40–50% in the CKD population compared to 2–3% in the general population [18]. Moreover, an episode of hyperkalemia in patients with CKD increases the odds of mortality within one day of the event [19]. In our population, the presence of hyperkalemia increased the risk of death by 54% and the risk of dialysis by 216%. However, since the relationship between hyperkalemia and the progression of CKD to dialysis is only the result of a statistical association, do not necessarily imply a cause-effect relationship. While significant structural damage has long been demonstrated for hypokalemia [20], particularly at the level of the kidney tubule, little is known about the damage of hyperkalemia to renal tissue. However, even with regard to hyperkalemia, we can hypothesize chronic modifications of the kidney tissue that may promote the damage progress itself. CKD is characterized by a loss of nephron mass and a reduction in the number of collecting ducts to secrete K. The chronic nature of this process allows an adaptive response to occur in the remaining nephrons, thereby allowing the amount of K excreted per unit GFR (fractional excretion of K) to increase, which is due to the increased K secretory capacity resulting from structural changes that occur in the distal nephron and principal cells of the collecting duct. These changes are similar to those that occur in response to chronic K loading in normal subjects and include proliferation and in-folding of the basolateral membrane, cellular hypertrophy, and increased mitochondrial number [21]. Furthermore, hyperkalemia has also been associated with other diseases such as diabetes and heart failure that favor an advanced stage of CKD [12, 22]. The highest risk of hyperkalemia has been reported in patients with diabetes, advanced CKD, kidney transplant recipients, and patients treated with RAASi [20]. Treatment with RAASi, such as ACEi or ARBs, is widely used for managing CKD progression. However, RAASi therapy in CKD is linked with an increased risk of hyperkalemia, especially when ACEi and ARBs are administered in combination. Ninety-two per cent of our patients were established users of RAASi therapies. In a study of 1,448 randomly assigned patients with proteinuric diabetic nephropathy, Fried et al. [23] showed that the risk of hyperkalemia was more than double in the combination-therapy group (ACEi and ARBs) compared to the monotherapy group. That study was stopped early due to safety concerns [23]. In a cohort of patients with possible CKD who started an ACE inhibitor, Johnson et al. identified seven patient characteristics that predicted 90-day risk of hyperkalemia: advanced age (80–89 years), pre-existing declining kidney function, diabetes, heart failure, high starting dose of ACEi (ramipril > 10 mg/day), current use of potassium supplements, and current use of ARBs or potassium-sparing diuretics [24]. Of course, the higher the baseline serum potassium, the higher the risk of hyperkalemia with RAAS blockade therapy. However, the other risk predictor factors may also be useful for more intensive potassium monitoring and subsequent intervention in CKD patients on RAASi therapy. Therefore, ACEi and ARBs have demonstrated efficacy in reducing blood pressure and proteinuria, slowing progression of kidney disease, and improving outcomes in patients with heart failure, diabetes mellitus, and post–myocardial infarction. However, all these beneficial effects of RAASi could be threatened by the appearance of hyperkalemia. Indeed, hyperkalemia can play either a direct role in the progression of CKD and the occurrence of cardiovascular events, or an indirect role, consequent to discontinuation of RAAS inhibitors after hyperkalemia events [25].

In a recent study focusing on heart failure patients with hyperkalemia and a high frequency of CKD who were on RAASi therapy, the risk of all-cause death was found to be closely related to RAASi (ACEi, ARBs or mineralocorticoid receptor antagonists [MRAs]) discontinuation rather than to hyperkalemia levels themselves [26]. In our study, the increased risk of all-cause death and cardiovascular events is associated with non-adherence to RAASi treatment, with an HR of 1.45 for cardiovascular events and 2.26 for death. RAASi discontinuation rates due to hyperkalemia onset were very low (0.4–8.1%) in randomized trials in patients with heart failure [27] or hypertension [28]. Moreover, data on the discontinuation of RAASi therapy following hyperkalemia in CKD in routine clinical settings are few and limited. Our population-based study showed a RAASi discontinuation or sub-optimal adherence prevalence of 21.8% and 33.6% after hyperkalemia onset, respectively. Epstein [29] reported that among patients receiving RAASi therapy at the maximum dosage, 47% underwent dose reduction or therapy discontinuation following hyperkalemic events. Our results are consistent with Epstein’s data and suggest that RAASi dose titration is relatively common in routine clinical settings among CKD patients who experience hyperkalemia. Unfortunately, such changes in RAASi treatment regimens and non-adherence following hyperkalemia may have relevant consequences on the “trajectory” of future cardiovascular events and mortality. We observed an overall cardiovascular events incidence rate of 21.4 per 100 person-years in non-adherent patients, while in adherent patients, the incidence rate was 13.4. The incidence of death for all cause was 29.2/100 person-years in non-adherent and 10.8/100 person-years in adherent patients. These findings suggest that hyperkalemia may be a great limiting factor for optimal RAASi use in people with CKD, and that it could nullify the benefits obtained concerning cardiovascular diseases and death risk.

Our study was conducted on a large population-based cohort of patients with CKD reflecting real-world clinical settings. In addition, based on longitudinal data, we assessed the effects of hyperkalemia in RAASi users with CKD, medication adherence, and the consequences of non-optimal RAASi therapy on patient outcomes. However, our study has some limitations, mainly due to the descriptive nature of the analysis which is based on data collected through administrative databases. The first limitation is the lack of clinical information on co-morbidities or other potential confounders (e.g. proteinuria, systolic and diastolic blood pressure, disease severity) that could have influenced our results. Secondly, due to the nature of the data source it was not possible to capture CKD diagnoses outside hospitalizations; moreover, since potassium levels are more likely to be measured in patients at higher risk of dyskalemia, a selection bias may have occurred. Furthermore, administrative databases do not collect information pertaining to the cause of death or the reasons behind non-adherence, and CKD stage is not always correctly reported in the hospitalization databases. Therefore, we could have underestimated the proportion of patients in each stage. Another limitation concerns follow-up length, since patients had different follow-up times because they were enrolled at different times throughout the inclusion period. Finally, our study is retrospective and observational and we did not include a “control” group of persons with normal kidney function with whom the study outcomes could have been compared.

In conclusion, in a large cohort of adult RAASi users with CKD, we observed that following the onset of hyperkalemia a consistent proportion of previously adherent patients discontinued RAASi therapy or reported lower adherence. Unfortunately, non-adherence to RAASi medication can result in an increased risk of cardiovascular events and death. Furthermore, patients with hyperkalemia had a significantly increased risk of cardiovascular events, death and dialysis initiation.

Maintaining an optimal dosage of RAASi in populations at risk of CKD progression, heart failure, and diabetes while implementing various strategies to control potassium balance is desirable. The emergence of new drugs, aimed specifically at controlling serum potassium, could lead to preventive management of hyperkalemia in an effort to maintain the beneficial effects of the RAASi blockade unaltered.

## Supplementary Information

Below is the link to the electronic supplementary material.

Supplementary file1 (DOCX 14 KB)

Supplementary file2 (DOCX 16 KB)

Supplementary file3 (DOCX 14 KB)

## Data Availability

The datasets used and/or analyzed during the current study are available from the corresponding author on reasonable request.
