# Supplementary material for: Association between hyperkalemia, RAASi non-adherence and outcomes in chronic kidney disease
Source: J Nephrol. 2021 Jun 11;35(2):463–72. doi: 10.1007/s40620-021-01070-6 (PMC8927011; doi:10.1007/s40620-021-01070-6)
Supplement: Supplementary file 1 — Supplementary file1 (DOCX 14 KB) [file 40620_2021_1070_MOESM1_ESM.docx]

**Supplementary Table 1. Risk of cardiovascular events or death for nonadherent vs adherent**

**to RAASi patients (Cox adjusted model).***

|  | cardiovascular events | | Death | |
| --- | --- | --- | --- | --- |
|  | HR [95% CI] | P value | HR [95% CI] | P value |
| Adherence | 1 | - | 1 | - |
| Nonadherence | 1.35 [1.01-1.80] | 0.044 | 2.26 [1.71-2.99] | <0.001 |
| Age | 1.01 [1.00-1.02] | 0.010 | 1.05 [1.04-1.06] | <0.001 |
| Male gender | 1.46 [1.10-1.94] | 0.008 | 1.19 [0.95-1.50] | 0.129 |
| Charlson Comorbidity Index | 1.06 [0.98-1.14] | 0.128 | 1.20 [1.13-1.27] | <0.001 |
| CKD stage | 0.87 [0.67-1.14] | 0.326 | 0.96 [0.77-1.21] | 0.755 |

Abbreviation: CKD, chronic kidney disease; PSM, propensity score matching; HR, hazard ratio;

CI, confidence interval.* Due to the low sample size, only age, male gender,

Charlson Index and CKD stage were considered.
