# Supplementary material for: Association between hyperkalemia, RAASi non-adherence and outcomes in chronic kidney disease
Source: J Nephrol. 2021 Jun 11;35(2):463–72. doi: 10.1007/s40620-021-01070-6 (PMC8927011; doi:10.1007/s40620-021-01070-6)
Supplement: Supplementary file 2 — Supplementary file2 (DOCX 16 KB) [file 40620_2021_1070_MOESM2_ESM.docx]

**Supplementary Table 2. Risk of cardiovascualr events, death, or dialysis for Hyperkalemia vs Non-hyperkalemia patients (Cox adjusted model).**

|  | CV events | | Death | | Dialysis | |
| --- | --- | --- | --- | --- | --- | --- |
|  | HR [95% CI] | P value | HR [95% CI] | P value | HR [95% CI] | P value |
| Non-hyperkalemia | 1 | - | 1 | - | 1 | - |
| Hyperkalemia | 1.31 [1.13-1.51] | <0.001 | 1.70 [1.48-1.95] | <0.001 | 3.43 [2.59-4.55] | <0.001 |
| Age | 1.03 [1.02-1.03] | <0.001 | 1.07 [1.06-1.08] | <0.001 | 0.97 [0.96-0.98] | <0.001 |
| Male Gender | 1.39 [1.22-1.59] | <0.001 | 1.08 [0.95-1.24] | 0.225 | 1.00 [0.77-1.31] | 0.987 |
| Charlson Comorbidity Index | 1.02 [0.98-1.07 | 0.305 | 1.19 [1.14-1.24] | <0.001 | 0.89 [0.79-0.99] | 0.036 |
| CKD stage | 1.11 [0.96-1.29] | 0.162 | 1.48 [1.27-1.71] | <0.001 | 7.21 [5.13-10.12] | <0.001 |
| Previous hospitalization related to CKD | 1.02 [0.76-1.37] | 0.895 | 0.94 [0.70-1.27] | 0.685 | 2.07 [1.38-3.10] | <0.001 |
| **Previous drug treatments** |  |  |  |  |  |  |
| RAASi use | 1.00 [0.77-1.31] | 0.979 | 0.80 [0.62-1.03] | 0.078 | 0.73 [0.48-1.11] | 0.139 |
| Diuretics (%) | 1.08 [0.94-1.24] | 0.266 | 1.35 [1.16-1.56] | <0.001 | 0.98 [0.73-1.32] | 0.893 |
| Aldosterone antagonists | 1.11 [0.91-1.36] | 0.287 | 1.23 [1.02-1.48] | 0.029 | 0.65 [0.37-1.12] | 0.121 |
| Beta blocking agents | 1.32 [1.16-1.50] | <0.001 | 0.96 [0.84-1.09] | 0.518 | 1.04 [0.79-1.38] | 0.763 |
| Lipid modifying agents | 1.51 [1.32-1.73] | <0.001 | 0.79 [0.69-0.90] | 0.001 | 1.21 [0.91-1.61] | 0.187 |
| Antidiabetics | 1.35 [1.16-1.56] | <0.001 | 0.96 [0.83-1.11] | 0.572 | 1.40 [0.99-1.98] | 0.059 |
| RAASi dosage | 1.00 [1.00] | 0.007 | 1.00 [1.00] | 0.284 | 1.00 [1.00] | 0.868 |
