# Supplementary material for: Association between hyperkalemia, RAASi non-adherence and outcomes in chronic kidney disease
Source: J Nephrol. 2021 Jun 11;35(2):463–72. doi: 10.1007/s40620-021-01070-6 (PMC8927011; doi:10.1007/s40620-021-01070-6)
Supplement: Supplementary file 3 — Supplementary file3 (DOCX 14 KB) [file 40620_2021_1070_MOESM3_ESM.docx]

**Supplementary Table 3. Risk of cardiovascular events or death for nonadherent vs adherent**

**to RAASi Non-hyperkalemia patients after PSM.***

|  | cardiovascular events | | Death | |
| --- | --- | --- | --- | --- |
|  | HR [95% CI] | P value | HR [95% CI] | P value |
| Adherence | 1 | - | 1 | - |
| Nonadherence | 1.11 [0.93-1.32] | 0.252 | 1.29 [1.07-1.56] | 0.009 |
| Age | 1.02 [1.01-1.03] | <0.001 | 1.06 [1.05-1.07] | <0.001 |
| Male gender | 1.34 [1.11-1.61] | 0.002 | 1.18 [0.97-1.44] | 0.097 |
| Charlson Comorbidity Index | 1.06 [1.00-1.11] | 0.032 | 1.17 [1.11-1.23] | <0.001 |
| CKD stage | 1.19 [0.95-1.47] | 0.125 | 1.66 [1.33-2.07] | <0.001 |
